# Affective States, Coping and Mutual Understanding in Russian Families During the Coronavirus Disease 2019 Pandemic Lockdown

**DOI:** 10.3389/fpsyg.2021.647029

**Published:** 2021-06-16

**Authors:** Elena V. Leonova, Alexey V. Khavylo

**Affiliations:** General and Legal Psychology Department, Tsiolkovskiy Kaluga State University, Kaluga, Russia

**Keywords:** COVID-19, lockdown in Russia, stress, coping, positive and negative affect, well-being, mutual understanding, parents and children

## Abstract

The purpose of the empirical study (April–May 2020) was to determine the type and level of affect, specifics of coping styles during the coronavirus disease 2019 (COVID-19) pandemic lockdown, as well as mutual understanding between parents and children. We hypothesized that the combination of positive and negative affect magnitude is a factor in well-being and mutual understanding with children, as well as the coping style during the lockdown. The study involved 705 respondents aged 16–77, including 435 parents living with children under 16. Personal traits, positive and negative affect, coping styles, and well-being were studied by Ten-Item Personality Inventory (TIPI)-RU, Positive and Negative Affect Scale (PANAS)-RU, Brief COPE, Satisfaction with Life Scale (SWLS), respectively. Mutual understanding was studied using a self-report questionnaire. Cluster analysis (k-means method) was used to divide the sample into clusters in accordance with the combination of positive and negative affect. According to the data obtained, parents from the “positive-affective” cluster have much better mutual understanding with both younger and older children than participants from other clusters.

## Introduction

The coronavirus disease 2019 (COVID-19) pandemic is a global challenge to humanity. According to the website of the Center for Systems Science and Engineering (CSSE) of Johns Hopkins University (Johns Hopkins Coronavirus Resource Center, 2021), more than 154 million cases of COVID-19 infection have been officially reported worldwide in 192 countries. The total number of deaths in May 2021 was more than 3.2 million people. The proliferation of COVID-19 can be considered a psychotraumatic situation characterized by several distinctive features (Bykhovets and Cohen-Lerner, 2020). New evidence suggests that the incidence of post-traumatic stress and psychological stress in the general population is increasing due to COVID-19 (Cooke et al., 2020).

Psychological stressors include lifestyle changes associated with imposed restrictions, switching to remote forms of work and studying; economic difficulties caused by the lockdown; informational impact from the media; lack of understanding of the people about the nature of the viral infection; and the mechanisms of its spread.

Changes in the socio-economic sphere are another significant source of psychological stress. Delay or reduction of wages, actual job loss or risk of losing it, forced unpaid leave, suspension or loss of business of an individual act as sources of deep emotional experiences and increase stress levels.

From the perspective of modern ecological immunology, the well-being of an organism is maintained by efficiently matching biological and behavioral priorities to the demands of the environment. The data on the influence of the number and intensity of social contacts on the level of immunity are quite contradictory (Segerstrom, 2010). On the one hand, epidemiological evidence correlating fewer social networks with increased all-cause mortality supports the idea that social relationships buffer against stress and improve health (House et al., 1988). On the other hand, extensive social contacts have been associated with poorer cellular immunity in healthy young adults and patients with HIV (Miller and Cole, 1998; Segerstrom, 2008). However, social network size was either unrelated to immunity and health or had negative consequences, particularly in prospective studies (Segerstrom, 2010). For certain categories, social support is especially important. For example, availability of social support leads to later symptoms of HIV infection onset and longer survival (Miller and Cole, 1998). So, the perceived social support (especially within the family) plays a special role.

There were additional stressors in the families with school-age children during the COVID-19 pandemic lockdown. They include the constant joint stay at home (in a limited space) of all family members as well as the need for parents to independently organize the education and leisure of children. Not all families had enough gadgets and satisfactory Internet connection for full-fledged distant work and study at the same time, which was an additional stress factor for families with children. In many families, parent-child relations worsened during the COVID-19 lockdown (Hiraoka and Tomoda, 2020). High levels of anxiety and depressive symptoms are associated with higher child abuse potential (Brown et al., 2020).

In accordance with Hobfoll’s Conservation of Resources Theory (COR) psychological stress is defined as a reaction to the environment in which there is (a) a threat of a net loss of resources, or (b) a lack of resource gain following the investment of resources Hobfoll (1989). Both perceived and actual loss or lack of gain are envisaged as sufficient for producing stress (Hobfoll, 1989). During the COVID-19 lockdown, almost everyone had decreased resources, both material, physical, and psychological, which led to severe stress. It should be noted that any person during their life has developed well-established patterns of reaction to stress (coping styles).

Various approaches and models of human stress response have been developed now. Categories and systems used to classify coping have been developed (Skinner et al., 2003). In accordance with the Carver approach 14 coping styles are distinguished: Active Coping, Planning, Suppression of Competing Activities, Restraint Coping, Seeking Social Support (instrumental and emotional reasons), Positive Reinterpretation and Growth, Acceptance, Turning to Religion, Focus on and Venting of Emotions, Denial, Behavioral Disengagement, Mental Disengagement, Alcohol-Drug Disengagement (Carver et al., 1989). Some of these styles (planning, positive reinterpretation, etc.) can be categorized as stress overcome resources leading to personal development, better understanding in the family, positive emotional mood, and well-being. That is why we suggested the relationship between coping styles, affect the magnitude, well-being, and mutual understanding between parents and their children.

Parenting behaviors cannot be fully understood without considering the emotional dysregulation of parents and their emotional regulation strategies (Barros et al., 2015); their overall subjective emotional well-being is a cause of somatic and mental health as well as success in various areas of life (Diener and Tay, 2017; Diener et al., 2018).

To measure subjective well-being, the model of Diener uses combination indicators of positive and negative emotions, such as the Positive and Negative Affect Scale (PANAS; Watson et al., 1988) or the Positive and Negative Experiences Scale (SPANE; Diener et al., 2009), and life satisfaction, such as the Life Satisfaction Scale (SWLS; LSS; Diener et al., 1985). Productive coping styles (as opposed to destructive coping styles) also promote subjective emotional well-being, one of the factors of mutual understanding in the family (Kryukova et al., 2019).

During the year 2020, research groups around the world have conducted numerous studies on the psychological effects of the COVID-19 pandemic. The totality of these studies, performed in different countries, at different times, on different samples, presents a very complex mosaic. It will take some time for the scientific community to comprehend the results of these multiple and diverse studies. The purpose of this study is to add to the scientific evidence on mutual understanding in families during the COVID-19 lockdown.

So, we hypothesized that the combination of the positive and negative affect magnitude of parents influences their well-being and mutual understanding with children, as well as their coping style during the COVID-19 lockdown.

The purpose of the empirical study was to determine the type and level of affect, specifics of coping with stress during the pandemic lockdown, as well as mutual understanding between parents and children.

## Materials and Methods

This study was carried out in accordance with the recommendations of the Declaration of Helsinki and the Ethical Committee of the Russian Psychological Society.

Data were collected in May 2020. In the study, we conducted two different cross-sectional surveys. There were 705 adult respondents in the combined sample: 597 female, age 36.52 ± 0.40, and 108 male, age 37.49 ± 1.15 (Khavylo and Leonova, 2020). The sample involved 435 parents (51 men, 384 women) living with children under 16. The surveys were completely anonymous and conducted online (Google forms and open-source application «1KA»); the responses of the individual participants were confidential. Participants received an invitation to participate and an informed consent form through both the educational online platform Network City (Kaluga region) and social networks (Facebook, VKontakte). Participants did not receive the remuneration for participation in the study.

Personal traits, positive and negative affect, coping was studied by Russian versions of well-known tests.

We have used the Ten-Item Personality Inventory (TIPI), which is a brief assessment of the Big-Five personality dimensions: (1) Extraversion, (2) Agreeableness, (3) Conscientiousness, (4) Emotional Stability, and (5) Openness to Experience (Gosling et al., 2003; Sergeeva et al., 2016). The Big-Five framework has become the most widely used and extensively researched model of personality (Costa and McCrae, 1992; John and Srivastava, 1999). The Big-Five framework is a hierarchical model of personality traits with five factors, which represent personality at the broadest level of abstraction. Each bipolar factor summarizes several more specific facets, which, in turn, subsume a large number of even more specific traits. The Big-Five framework suggests that most individual differences in human personality can be classified into five broad, empirically derived domains.

We have applied the PANAS-RU that measures two main aspects: positive and negative affect. Positive affect (PA) reflects the degree of activity, enthusiasm, and alert of a person. High PA is a state of high energy, concentration, and pleasure, while low PA is characterized by sadness and lethargy (Watson and Tellegen, 1985; Watson et al., 1988; Osin, 2012). Negative affect (NA) is a state of general distress and unpleasant interaction, with a low NA level reflecting calmness and serenity. Cronbach’s alpha coefficients for the current study were for PA scale 0.92, for NA scale 0.91.

Also, we have used the Brief COPE (Carver, 1997; Kryukova et al., 2019), which is a 28-item multidimensional measure of strategies used for coping or regulating cognitions in response to stressors. This abbreviated inventory (based on the complete 60-item COPE Inventory) is comprised of items that assess the frequency with which a person uses different coping styles. There are 14 two-item subscales within the Brief COPE, and each is analyzed separately: (1) Self-distraction, (2) Active Coping, (3) Denial, (4) Substance Use, (5) Use of Emotional Support, (6) Use of Instrumental Support, (7) Behavioral Disengagement, (8) Venting, (9) Positive Reframing, (10) Planning, (11) Humor, (12) Acceptance, (13) Religion, and (14) Self-blame. Cronbach’s alpha coefficients for the current study ranged between 0.38 and 0.85 for all aforementioned subscales of Brief COPE.

To measure satisfaction with life, we have used the SWLS (Diener et al., 1985; Osin and Leont’ev, 2008). It is a short five-item instrument designed to measure global cognitive judgments of satisfaction with the life of an individual. The SWLS was developed to assess satisfaction with the lives of people as a whole. The scale does not assess satisfaction with specific life domains, such as health or finances, but allows subjects to integrate and weigh these domains in whatever way they choose. Cronbach’s alpha coefficients for the SWLS scale for the current study were 0.89.

Mutual understanding between parents and their children (older and younger separately) was assessed by themselves *via* a five-point Likert scale (from 0 to 4): 0, no mutual understanding, frequent conflicts; 1, there is no mutual understanding, but conflicts are rare; 2, continuous conflicts and reconciliations; 3, mutual understanding in general, conflicts are rare; and 4, complete mutual understanding. We asked parents to assess their mutual understanding with both older and younger children twice: before COVID-19 lockdown (retrospectively) and during spring lockdown.

Survey participants with children were slightly older, but the average age difference was less than 3 years, which allows us to consider these groups as homogeneous in age.

## Results

Data analysis includes a comparative analysis (Student’s *t*-test) of coping styles and personality traits (among respondents with and without children) and Cluster analysis (k-means method) to divide the sample into groups with a similar ratio of positive and negative affect and subsequent comparison of the indicators of mutual understanding with children and coping styles in these groups. Statistica v.13 and SPSS v.26 software were used for computations.

At the first stage of data analysis, a comparative analysis of coping styles and personality traits among respondents with and without children was carried out.

To assess the significance of differences between the groups of respondents, the Student’s *t*-test was used. The respondents with children have significantly lower scores of PA (*p* = 0.011) and such coping styles as Self-distraction (*p* = 0.002), Behavioral Disengagement (*p* = 0.047), and Acceptance (*p* = 0.002). On the other hand, this group has higher scores of Active Coping (*p* = 0.022), Denial (*p* = 0.025), and Positive Reframing (*p* = 0.034). Comparative analysis revealed differences in individual personality traits among respondents with and without children. Respondents with children had higher scores on Conscientiousness (*p* = 0.004), Agreeableness (*p* = 0.051), Extraversion (*p* = 0.053), and lower scores on Openness to Experience (<0.001).

**Figure 1:**
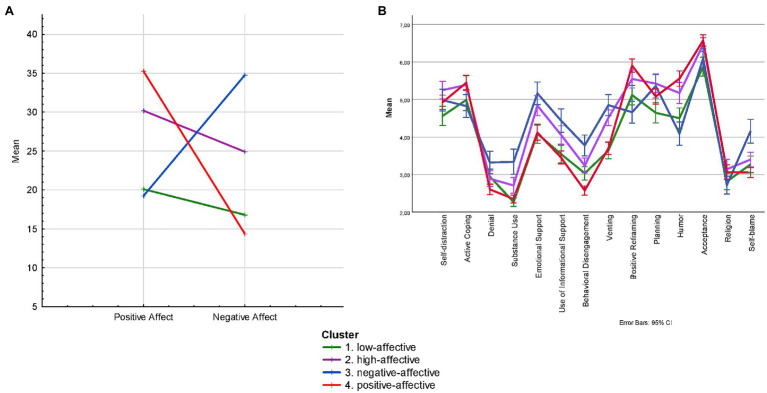
Mean values for each Cluster **(A)** and profile of coping styles in the Cluster **(B)**.

At the second stage of data analysis, to test the hypothesis that the combination of positive and negative affect magnitude is a factor in well-being and mutual understanding with children, we divided the sample into four clusters in accordance with the severity of positive and negative affect (Figure 1A). The division into clusters was done using the k-means method. The cluster extraction criterion was Fisher’s *F*-test (F_PA_ = 495,3∗∗; F_NA_ = 825,8∗∗).

Cluster 1 included respondents (*n* = 169) with relatively low scores on the scales of PA and NA. This group received a conditional name “low-affective.” Cluster 2 included respondents (*n* = 155) with higher scores compared to cluster 1 on the scales of PA and NA. This group received the conditional name “high-affective.” Participants in the survey with high scores on the NA scale and relatively low scores on the PA scale were included in cluster 3 (*n* = 121). This group was conditionally named “negative-affective.” Finally, the survey participants with high PA scores and relatively low PA scores comprised cluster 4 (*n* = 260). This group was conditionally named “positive-affective.” The *t*-test showed that all clusters except cluster 3 are characterized by a significant predominance of PA over Negative (*p* < 0.001).

In the next stage, we compared the selected groups of participants according to such indicators as coping styles used, personality traits, and features of relations with children. The statistical significance of differences between clusters was assessed using ANOVA. The general profile of coping styles is similar in all clusters (Figure 1B).

“Positive-affective” parents often use Positive Reframing, Humor, Acceptance (along with high-affective), humor and as well as, like “high-affective” parents, Humor and Active Coping. These parents are characterized by higher scores on the Openness to Experience, Conscientiousness, Extraversion, Agreeableness and Emotional Stability scales. According to the data obtained, “positive-affective” parents have much better mutual understanding with both younger and older children than parents from other clusters (Table 1).

**Table 1:** Mutual understanding with older and younger children: differences between clusters of parents, *t*-test absolute values.

Cluster number		1. Low-affective		2. High-affective		3. Negative-affective	
		Oldest	Youngest	Oldest	Youngest	Oldest	Youngest
1. Low-affective	Oldest						
	Youngest						
2. High-affective	Oldest	0.68					
	Youngest		0.34				
3. Negative-affective	Oldest	0.45		1.02			
	Youngest		0.15		1.17		
4. Positive-affective	Oldest	2.92^∗∗^		2.01^∗∗^		3.05^∗∗^	
	Youngest		2.85^∗∗^		2.25^∗∗^		2.63^∗∗^

∗∗ p < 0.01.

“Negative-affective” parents tend to use coping styles such as Denial, Substance Use, Use of Informational Support, Behavioral Disengagement, Venting, Self-blame. Parents in this group have lower Extraversion and Emotional Stability scores than other respondents. It can be assumed that the reduced affect is largely due to the personality traits of the representatives of this cluster.

“High-affective” parents are more likely to use Self-distraction and Planning (along with “negative-affective” parents).

Those parents who were combined in a “low-affective” cluster are less likely to use coping styles, especially such as Self-distraction and Planning.

Thus, we can say that the ratio of positive and negative affect during the pandemic is interconnected with the coping styles used. Probably, to a certain extent, the ways of reaction are conditioned by the expression of certain personality traits. Parents with a strong predominance of PA have better relationships with their children of different ages during the COVID-19 pandemic.

## Discussion

High PA can be defined as a state of pleasant engagement, high energy, and total concentration as opposed to dejection and lethargy (low PA). High NA corresponds to subjectively experiencing suffering and unpleasant involvement (variously anger, disgust, contempt, guilt, fear, and irritability) vs. calm and serenity (low NA).

According to numerous studies, NA scores correlate with the experience of stress and difficulties in coping with it, with the frequency of unpleasant life events, and with neuroticism (Watson et al., 1988). In turn, PA scores correlate with the frequency of pleasant events, extraversion, social engagement, close relationships, and measures of religiosity and spirituality (Watson, 2002). This agrees well with the results obtained in this study.

According to results, the respondents with children have significantly lower scores of PA compared to respondents without children. Though respondents with children are more conscientious, friendly, extraverted, they are less open to a new experience, as known, monotony does not contribute to emotional uplift. This is due, in our opinion, to the lesser possibility of emotional relief and relaxation in families with children during quarantine.

According to Banou et al. (2009), having a traumatic experience mediated through a decrease in available interpersonal resources increases susceptibility to psychological distress. On this basis, it can be assumed that traumatic relationships between children and their parents, the experience of family violence (and the observation of its manifestations between parents) during the pandemic may lead to a decrease in the level of available interpersonal resources in the long term.

The respondents with children rarely use coping styles as Self-distraction, Behavioral Disengagement, and Acceptance. On the other hand, this group has higher scores of Active Coping, Denial, and Positive Reframing. The reason for such results may be added responsibility of caring for children. This imposes restrictions on the use of certain coping styles.

Dividing the sample of parents into four categories according to the combination of the severity of positive and negative affect allowed us to test the hypothesis that the combination of the positive and negative affect magnitude of parents is influenced by their well-being and mutual understanding with children, as well as the coping style. It turned out that parents from the “positive-affective” cluster frequently use Positive Reframing, Humor, Acceptance, and, like the participants from the “high-affective” cluster, Active Coping. These people are characterized by higher scores on the Openness to Experience, Conscientiousness, Extraversion, Agreeableness, and Emotional Stability scales. As noted above, high scores on these scales can act as favorable psychological conditions for building good relationships with their children. According to the data obtained, people in this group have much better mutual understanding with both younger and older children than participants from other clusters.

The results of the study showed the role of positive emotions for general well-being, mutual understanding in families, avoidance of destructive coping styles during COVID-19 lockdown when many families were in a difficult situation. This is consistent with the results we obtained earlier (Leonova, 2020). On the other hand, the results confirm that mutual understanding can be considered as one of the resources in stressful situations (Pięta et al., 2019).

The research results help to understand the directions of psychological assistance and self-help for mutual understanding with children in conditions of limitations. Resource constraints do not affect the relationship directly. By choosing positive coping styles, it is possible to relieve the tension of an individual and not worsen relations with children.

We should note the following limitations of this study.

1. Only parents took part in the study (mostly mothers). It may be interesting to study mutual understanding from several points of view: both parents and each of their children in different living conditions.
2. Regional specificity of the results, most respondents live in Russian small and middle cities with a mild isolation regimen during the lockdown.
3. Mutual understanding before the lockdown was assessed retrospectively. Most of the participants that completed this survey, however, agreed to participate in a subsequent survey. In the future, we plan to conduct this study longitudinally. We believe that these efforts will help parents to correct their coping styles and increase mutual understanding with their children.

## Data Availability Statement

The dataset, called COVID19_Kaluga_RUS (Khavylo and Leonova, 2020) is deposited at https://doi.org/10.6084/m9.figshare.14229278 and has SPSS 22 format. The variables are described inside the dataset.

## Ethics Statement

Ethical approval was not provided for this study on human participants because Ethical standards in psychological studies in Russia are not legally accepted, but there are recommendations of the Ethics Committee of the Russian Psychological Society, which are consistent with the Declaration of Helsinki, and these recommendations were followed by us. The patients/participants provided their written informed consent to participate in this study.

## Author Contributions

The research results help to understand the directions of psychological assistance and self-help for mutual understanding with children in conditions of limitations. Resource constraints do not affect the relationship directly. By choosing positive coping strategies, it is possible to relieve one’s own tension and not worsen relations with children. All authors contributed to the article and approved the submitted version.

### Conflict of Interest

The authors declare that the research was conducted in the absence of any commercial or financial relationships that could be construed as a potential conflict of interest.
